# THE ROLE OF EDUCATION ON BRAIN PATHOLOGY AND COGNITIVE DECLINE ACROSS WOMEN AND MEN IN THE US

**DOI:** 10.1093/geroni/igae098.0595

**Published:** 2024-12-31

**Authors:** Min Hee Kim, Brittany Morin, Alice Olvera, Gelan Ying, Aayush Khadka, Seo-Eun Choi

**Affiliations:** Rutgers University, San Francisco, California, United States; University of California San Francisco, San Francisco, California, United States; Queens College at the City University of New York, New York, New York, United States; University of Florida, Gainesville, Florida, United States; University of California San Francisco, San Francisco, California, United States; University of Washington, Seattle, Washington, United States

## Abstract

Women have a higher risk of developing Alzheimer’s disease and related dementia (ADRD) than men. Education, as a key contributor to cognitive reserve, might protect the ADRD onset. While existing evidence suggests that increased educational attainment may attenuate the negative impact of neuropathology on cognitive function, these studies focused primarily on average cognitive function, and little is known about whether education similarly attenuates the relationship between neuropathology and cognitive decline among those with high or low levels of cognitive outcomes or sex/gender. Using the Alzheimer’s Disease Neuroimaging Initiative sample (772 male and 627 female participants, who were cognitively normal or had mild cognitive impairment at baseline), we investigated education attainment modified the association between anatomical reduction in the brain and cognitive decline in sex-stratified models. We applied quantile regression with a three-way interaction term (education x cortical thickness x years) against 10-year cognitive function, an established specification for testing the cognitive reserve mechanism. We included a robust set of biological and contextual confounders. Primary results showed that, among males, education had attenuated the relationship between brain pathology and cognitive decline at lower levels of cognitive function distribution. Among females, education attenuates brain pathology and cognitive decline in all levels of outcome distribution with large confidence intervals. We will discuss potential explanations for differential effect modification by education on the brain pathology-cognition relationship across sex/gender and expand its implications for future research and policy/practice intervention.

